# Melatonin Deficiency Confers Tolerance to Multiple Abiotic Stresses in Rice via Decreased Brassinosteroid Levels

**DOI:** 10.3390/ijms20205173

**Published:** 2019-10-18

**Authors:** Ok Jin Hwang, Kyoungwhan Back

**Affiliations:** Department of Biotechnology, College of Agricultural and Life Sciences, Chonnam National University, Gwangju 61186, Korea; smilax@jnu.ac.kr

**Keywords:** abiotic stress, brassinosteroid, cadmium, cold, gibberellin, heat, melatonin, salt

## Abstract

Melatonin has long been recognized as a positive signaling molecule and potent antioxidant in plants, which alleviates damage caused by adverse conditions such as salt, cold, and heat stress. In this study, we found a paradoxical role for melatonin in abiotic stress responses. Suppression of the serotonin *N*-acetyltransferase 2 (*snat2*) gene encoding the penultimate enzyme in melatonin biosynthesis led to simultaneous decreases in both melatonin and brassinosteroid (BR) levels, causing a semi-dwarf with erect leaf phenotype, typical of BR deficiency. Here, we further characterized *snat2* rice in terms of grain morphology and abiotic stress tolerance, to determine whether *snat2* rice exhibited characteristics similar to those of BR-deficient rice. As expected, the *snat2* rice exhibited tolerance to multiple stress conditions including cadmium, salt, cold, and heat, as evidenced by decreased malondialdehyde (MDA) levels and increased chlorophyll levels, in contrast with *SNAT2* overexpression lines, which were less tolerant to stress than wild type plants. In addition, the length and width of grain from *snat2* plants were reduced relative to the wild type, which is reminiscent of BR deficiency in rice. Other melatonin-deficient mutant rice lines with suppressed BR synthesis (i.e., *comt* and *t5h*) also showed tolerance to salt and heat stress, whereas melatonin-deficient rice seedlings without decreased BR levels (i.e., *tdc*) failed to exhibit increased stress tolerance, suggesting that stress tolerance was increased not by melatonin deficiency alone, but by a melatonin deficiency-mediated decrease in BR.

## 1. Introduction

Since melatonin was first identified in plants in 1995 [1,2], a growing body of research suggests that it plays roles as both a pleiotropic signal molecule and a potent antioxidant molecule in plants [3,4], similar to its reported roles in animals [5,6]. Early studies showed that treatment of plants with exogenous melatonin produced diverse physiological effects including high temperature tolerance [7], early flowering [8], cold tolerance [9,10], coleoptile and root growth [11,12], and salt tolerance [13]. Increased levels of endogenous melatonin were first produced in plants through ectopic overexpression (OE) of the serotonin *N*-acetyltransferase (*SNAT*) gene from *Chlamydomonas reinhardtii* in transgenic tomato [14], and subsequently by OE of the human *SNAT* and *N*-acetylserotonin *O*-methyltransferase (*ASMT*) genes in transgenic tobacco [15]. Of note, the transgenic tobacco plants exhibited enhanced antioxidant capacity in concert with simultaneous increases in the levels of antioxidant enzymes and the antioxidant glutathione, consistent with the effects of exogenous melatonin treatment described above.

**Figure 1:**
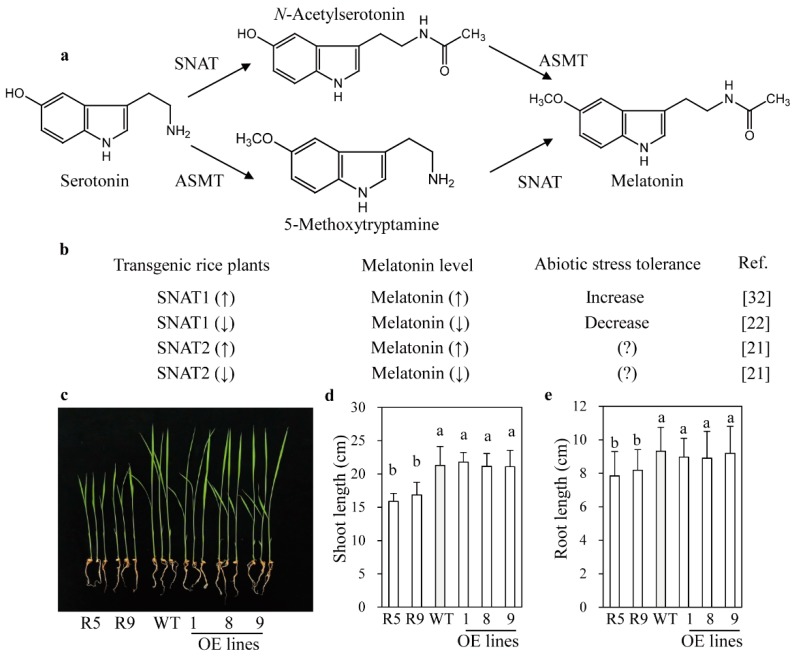
Phenotypes of *snat2* and *SNAT2* OE rice seedlings. (**a**) Melatonin biosynthetic pathway in plants. (**b**) Relationship between *SNAT* gene expression and stress tolerance. (**c**) Seedling phenotypes. (**d**) Shoot length. (**e**) Root length. Germinated seeds were grown under a 14-h light/10-h dark cycle for 12 days. Values are means ± standard deviation from three independent experiments. Different letters indicate significant differences, as determined by a post-hoc Tukey’s honestly significant difference (HSD) test at *p* < 0.05. R5 and R9: *SNAT2* RNAi lines; OE, *SNAT2* overexpression lines; WT, wild type. ↑ denotes increase and ↓ means decrease on melatonin level. ? denotes no data available.

SNAT and ASMT are the final two enzymes in the melatonin biosynthetic pathway in plants (Figure 1a), and are responsible for the synthesis of *N*-acetylserotonin and melatonin, respectively [16]. Plant *ASMT* genes were first cloned from rice in 2013 [17], followed by the cloning of the *SNAT1* gene in 2013 [18] and the *SNAT2* gene in 2016 [19]. Serotonin is the only precursor used in the synthesis of melatonin. It is derived from tryptophan by two successive enzymatic reactions carried out by tryptophan decarboxylase (TDC) and tryptamine 5-hydroxylase (T5H). Although the above four enzymes are crucial for melatonin biosynthesis in plants, SNAT appears to be the pivotal enzyme because it is highly conserved from cyanobacteria to land plants [20] and is present as at least two distinct isogenes that share very low amino acid homology [19]. The most prominent phenotype caused by melatonin depletion resulting from the disruption of melatonin biosynthetic pathway genes is a semi-dwarf with erect leaf phenotype, which was recently observed in *SNAT2* suppression (*snat2*) rice lines [21]. By contrast, no such phenotype was observed in *SNAT1* suppression rice lines [22]. A *T5H* knockout mutant (*t5h*) rice line and a caffeic acid *O*-methyltransferase (*COMT*) suppression (*comt*) rice line also exhibited semi-dwarf with erect leaf, brassinosteroid (BR)-deficient phenotypes comparable to that of *snat2* rice through the suppression of melatonin synthesis due to the high ASMT activity of COMT [23]. Alternatively, serotonin can be converted into 5-methoxytryptamine (5MT) by ASMT or COMT, followed by melatonin synthesis by SNAT (Figure 1a) [24]. Due to its high *K*_m_ toward serotonin by ASMT, serotonin-5MT-melatonin pathway may occur under stress conditions whereas serotonin-NAS-melatonin pathway works under normal growth conditions [16].

In addition to the above effects of exogenous melatonin application or endogenous increase by ectopic OE, melatonin has also been implicated in germination, growth, and defense against abiotic and biotic stresses. An increase in melatonin is associated with enhanced resistance to stress, possibly due to direct and indirect effects of the potent antioxidant activity of melatonin [3,25]. Importantly, a recent report shed new light on the possible role of melatonin as a signaling molecule that regulates skotomorphogenesis, which is characterized by rapid hypocotyl growth in darkness [26]. A decrease in the endogenous melatonin level caused a decrease in BR levels, leading to a semi-dwarf with erect leaf phenotype typical of BR-deficient and BR-signaling mutants [21]. However, a melatonin decrease is not necessarily coupled with a BR decrease; BR decrease occurs in a melatonin biosynthetic gene-specific manner. The suppression of *T5H* (*t5h*), *SNAT2* (*snat2*), and *COMT* (*comt*) resulted in decreased BR levels, whereas suppression of *TDC* (*tdc*) and *SNAT1* (*snat1*) expression did not [23].

BR is a well-known plant hormone involved in skotomorphogenesis and abiotic stress tolerance in plants [27]. Exogenous application of BR confers protection against cold, salt, heat, and heavy metal stresses [28], as has also been shown for melatonin [25]. Paradoxically, some BR-deficient and BR-signaling mutants displayed tolerance to cold [29], salt [30], and drought [31], whereas BR OE mutants were more sensitive to the same stresses [29]. Therefore, we examined whether melatonin-deficient mutants exhibiting decreased BR levels are also tolerant to abiotic stress in a manner similar to BR mutants. In this study, we found that *snat2* rice plants producing decreased levels of melatonin and BR exhibited increased tolerance to a number of abiotic stresses, whereas *SNAT2* OE plants exhibited stress tolerance comparable to or less than that of the wild type. Moreover, melatonin-deficient *t5h* mutant and *comt* rice plants with decreased BR levels also exhibited stress tolerance similar to that of *snat2* rice.

## 2. Results

### 2.1. Phenotypes of snat2 and SNAT2 OE Rice Seedlings

The previously generated *snat2* rice line, in which *SNAT2* expression is suppressed by RNAi, produces low melatonin levels and exhibits a semi-dwarf with erect leaf phenotype in conjunction with decreased BR levels [21]. The *SNAT2* mRNA was reduced two-fold in the *snat2* whereas increased more than five-fold in *SNAT2* OE rice based on qPCR analyses. By contrast, the erect leaf phenotype was not observed in *snat1* rice line [22], which exhibits decreased melatonin levels and a lower level of tolerance to salt and cold than the wild type, while *SNAT1* OE rice plants produced higher melatonin levels, and exhibited significant tolerance to cadmium and delayed senescence (Figure 1b) [32]. Therefore, we examined whether *snat2* and *SNAT2* OE rice exhibited similar abiotic stress responses to *snat1* and *SNAT1* OE rice. When *snat2* and *SNAT2* OE rice seeds were germinated and grown in soil in pots for 12 days under a 14-h light/10-h dark cycle, the *snat2* seedlings exhibited shorter shoot and root lengths than wild-type seedlings, but no difference was observed between the *SNAT2* OE lines and the wild type (Figure 1c–e). The second leaf angles were significantly smaller in the *snat2* rice than in the wild type, while the *SNAT2* OE lines showed leaf angles comparable to those of the wild type [21]. Some *SNAT2* OE plants exhibited increased leaf angles relative to the wild type, but the differences were not statistically significant. These data indicated that *snat2* rice resembled BR-deficient rice, which is characterized by a dwarf and erect leaf phenotype, while *SNAT2* OE rice was dissimilar to BR-proficient rice with the increased leaf angle [33].

### 2.2. Grain Phenotypes and Yield Performance

**Figure 2:**
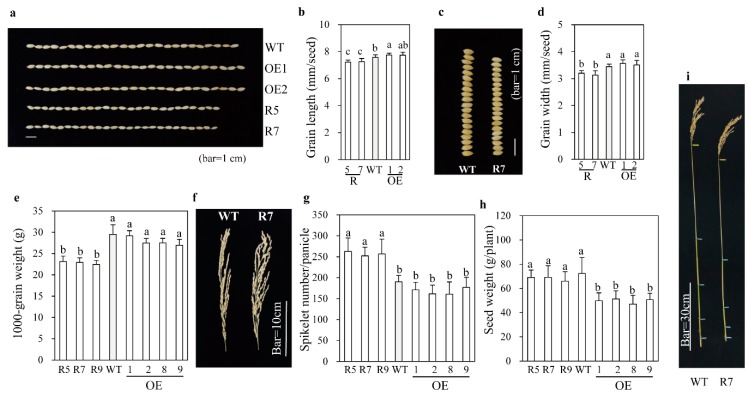
Rice grain and panicle morphology. (**a**) Photograph showing lengths of grains from WT, OE, and *snat2* lines. (**b**) Grain lengths (*n* = 200). (**c**) Photograph showing widths of grains from *snat2* line and WT. (**d**) Grain widths (*n* = 200). (**e**) 1000-grain weights (*n* = 4). (**f**) Representative panicle morphologies. (**g**) Spikelet numbers (*n* = 20). (**h**) Seed weights (*n* = 10). (**i**) Culm length of adult rice. T_2_ homozygous rice seeds were germinated and grown in a paddy field. Different letters indicate significant differences as determined by a post-hoc Tukey’s HSD test at *p* < 0.05.

Rice grain size is affected by BR levels, with BR-deficient rice plants producing small grains [34,35]. To verify whether grain size was affected in *snat2* rice, we investigated yield components of *snat2* rice grown in a paddy field. As shown in Figure 2, the grain length was longer in *SNAT2* OE plants than in the wild type, but was shorter in *snat2* plants than in the wild type (Figure 2a,b). Grain width was reduced in *snat2* plants, but no difference was observed between *SNAT2* OE and wild-type grains (Figure 2c,d). Due to the smaller grain size, the 1000-grain weight was less for *snat2* than for the wild type (Figure 2e). The rice inflorescence morphology differed significantly between the wild-type and *snat2* plants, with the *snat2* inflorescences possessing more primary branches resulting in higher spikelet numbers per plant (Figure 2f,g); meanwhile, the inflorescence morphology of *SNAT2* OE plants was similar to that of the wild type. The higher numbers of spikelets per plant in *snat2* successfully compensated for the reduced grain size, leading to no yield penalty relative to the wild type (Figure 2h). The adult *snat2* rice showed a semi-dwarf phenotype (Figure 2i). Increased spikelet number per panicle had also been observed in the *d61* rice BR receptor mutant [36], but not in the BR biosynthetic mutant, *dwarf4-1* [37]. The same 1000-grain weight in *SNAT2* OE with long grain length relative over wild type was attributed to the reduced thickness of *SNAT2* OE seed relative over wild type. These data suggest that, with respect to panicle number, the effects of *snat2*-mediated melatonin reduction are more closely associated with BR signaling than with BR deficiency.

### 2.3. Altered GA Sensitivity in snat2 Rice

**Figure 3:**
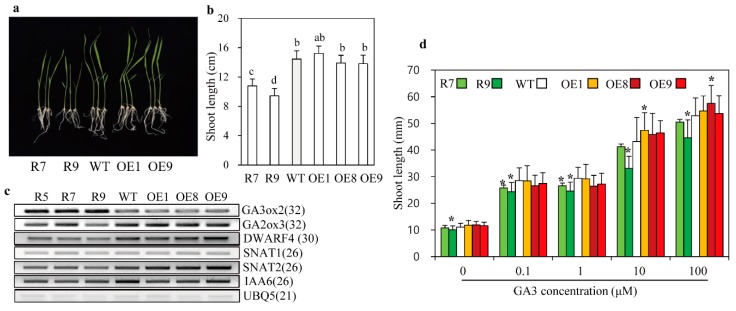
Gibberellic acid (GA) and brassinosteroid (BR) biosynthetic gene expression levels and GA sensitivity assay. (**a**) Phenotypes of various rice seedlings grown for 7 days. (**b**) Shoot lengths. (**c**) Expression levels of GA and BR biosynthetic genes determined by reverse transcription-polymerase chain reaction (RT-PCR). (**d**) Shoot lengths of GA-treated seedlings. Rice seedlings were grown for 7 days in half-strength MS medium (**a**–**c**). Rice seeds were grown at various concentrations of GA_3_ after pretreatment with the GA biosynthesis inhibitor paclobutrazol (PAC) (**d**). Different letters and asterisks indicate significant differences as determined by a post-hoc Tukey’s HSD test at *p* < 0.05. GenBank accession numbers are AB056519 (*GA3ox*2), AK101713 (*GA2ox3*), Os03g12660 (*DWARF*4), and Os03g13170 (*UBQ5*). Numbers in parentheses indicate the number of PCR cycles.

**Figure 4:**
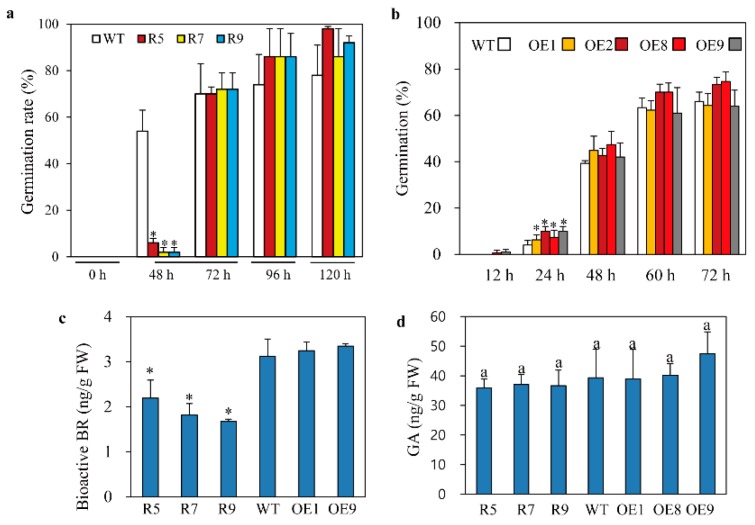
Germination rates and bioactive BR and GA levels. (**a**) Time course of *snat2* rice germination. (**b**) Time course of *SNAT2* OE rice germination. (**c**) Bioactive BR levels. (**d**) Bioactive GA levels. Rice seeds were germinated under 14-h light/10-h dark long day conditions for the germination test. BR and GA levels were quantified by ELISA using 7-day-old rice seedlings grown in half-strength MS medium. Different letters and asterisks indicate significant differences as determined by a post-hoc Tukey’s HSD test at *p* < 0.05. Data represent the mean ± standard deviation of triplicate experiments.

In plants, the dwarf phenotype results from a defect in either BR or GA signaling and biosynthesis, indicative of a close relationship between BR and GA [38]. In this context, the BR biosynthesis mutant, *d11*, produced lower GA levels than the wild type. To study the relationship between BR reduction and GA levels in *snat2* rice, we performed a GA-sensitivity assay. Seven-day-old *snat2* seedlings were shorter than wild-type and *SNAT2* OE seedlings (Figure 3a,b). Expression levels of the active GA biosynthetic gene, *GA3ox2*, were enhanced in *snat2*, whereas expression of the inactive GA biosynthetic gene, *GA2ox3*, was downregulated relative to that in wild-type and *SNAT2* OE plants (Figure 3c). As reported previously [21], the major BR biosynthetic gene, *DWARF4*, was expressed at lower levels in *snat2* plants, but in this study we observed no difference in its expression in *SNAT2* OE relative over wild-type (Figure 3c). Because grain phenotype and dwarfism can be easily connected to the relationship between BR and auxin [39], we also measured the expression levels of a representative auxin responsive gene *IAA6* [40]. The *IAA6* was downregulated in the *snat2* rice and *SNAT2* OE rice lines except OE9, suggestive of a possible involvement of auxin as well. The increase in *GA3ox2* expression may suggest an increase in GA levels or possible feedback regulation compensating for a decrease in GA in the *snat2* rice. In the presence of the GA inhibitor PAC, all rice seedlings showed similar shoot lengths of approximately 10 mm (Figure 3d). By contrast, addition of GA_3_ resulted in increased shoot length in a GA_3_ dose-dependent manner in all rice seedlings; however, the lengths of *snat2* seedlings were shorter than those of wild-type seedlings at all concentrations of GA_3_. Of note, the shoot lengths of the *SNAT2* OE seedlings appeared to increase under high concentrations of GA_3_, such as 10 and 100 μM, but the increase in shoot length varied depending on the lines (Figure 3d). These data indicate that *snat2*-mediated melatonin deficiency is closely linked to reduced BR levels when analyzed by ELISA method, as well as increased GA insensitivity. To further assess GA involvement, we performed a germination test under light/dark conditions. As shown in Figure 4, the germination rate of *snat2* rice was severely reduced at 48 h, suggesting increased GA insensitivity, whereas the germination rate of *SNAT2* OE rice was increased at 24 h, suggesting enhanced GA sensitivity. Overall germination rates after 72 h did not differ between the wild type and *SNAT2* OE (Figure 4a,b). The bioactive BR content was lower in *snat2* than in the wild type, while no significant difference in BR content was observed between *SNAT2* OE and the wild type (Figure 4c). GA content was unaltered in both *SNAT2* OE and *snat2* (Figure 4d). Thus, it can be concluded that the increase in GA biosynthetic gene expression was caused by feedback regulation, owing to decreased GA sensitivity. In summary, we suggest that the decreased melatonin levels in the *snat2* plants led to reduced BR levels, which increased GA insensitivity, while GA sensitivity appeared to increase slightly in the *SNAT2* OE rice lines.

### 2.4. Enhanced Tolerance to Cadmium Stress in Melatonin-Deficient snat2 Rice

**Figure 5:**
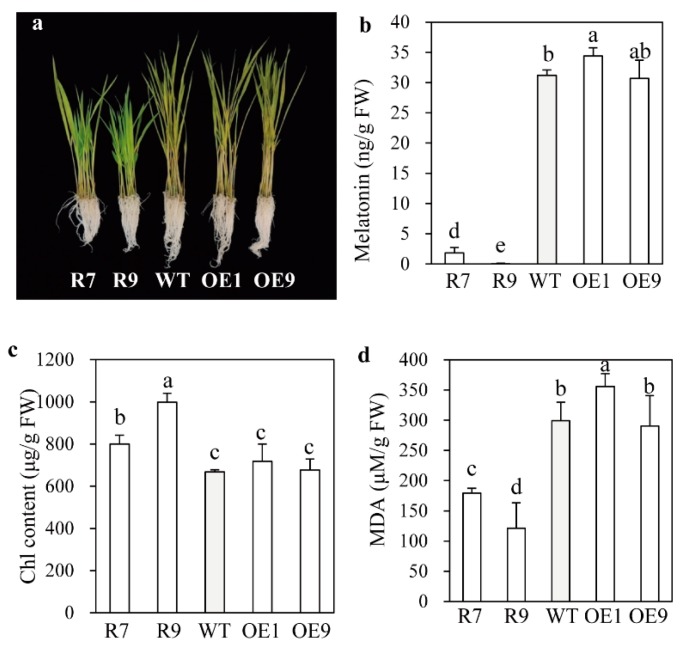
Effects of cadmium treatment on melatonin, chlorophyll, and malondialdehyde (MDA) levels in rice. (**a**) Rice seedlings after cadmium treatment. (**b**) Melatonin levels. (**c**) Chlorophyll content. (**d**) MDA content. Seven-day-old seedlings were challenged with cadmium (500 μM) for 3 days under a 14-h light/10-dark photoperiod. Shoot tissue was employed for analyses. Different letters indicate significant differences as determined by a post-hoc Tukey’s HSD test at *p* < 0.05.

Both reduced BR levels and increased GA insensitivity in *snat2* rice may result in the retarded seedling growth. These two factors have been known to enhance abiotic stress tolerance in plants [41,42]. Thus, we examined whether *snat2* rice plants exhibit tolerance to abiotic stress. We challenged rice seedlings with cadmium stress, which induces melatonin synthesis in rice [23]. As shown in Figure 5, melatonin levels were dramatically reduced in the *snat2* rice, whereas a slight increase was observed in the *SNAT2* OE lines relative to the wild type (Figure 5a,b). In contrast to many previous results in which melatonin was positively associated with enhanced abiotic stress tolerance [3,4,32], the melatonin-deficient *snat2* plants paradoxically showed higher tolerance to cadmium stress than the wild type, as evidenced by higher chlorophyll contents and lower MDA levels than in the wild type (Figure 5c,d). These results are attributed to the BR deficiency and increased GA insensitivity of *snat2* rice, and not to the removal of potential direct harmful effects of melatonin accumulation (since melatonin has no toxic effect in most plant species) [43]. As opposed to the *snat2*, the *SNAT2* OE exhibited less cadmium tolerance than wild type (Figure 5c,d).

### 2.5. Melatonin-Deficient snat2 Rice Plants Exhibit Resistance to Salt, Cold, and Heat Stress

**Figure 6:**
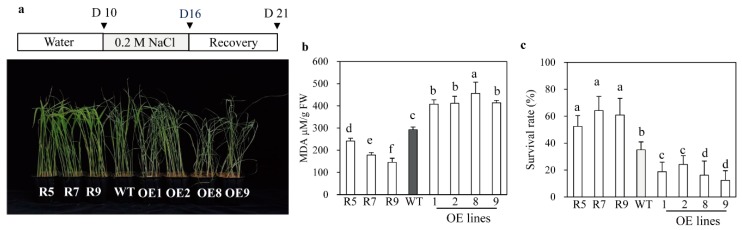
Response to salt treatment. (**a**) Rice seedlings after recovery from salt stress treatment. (**b**) MDA content. (**c**) Survival rates. Ten-day-old rice seedlings were immersed in 0.2 M NaCl solution for 6 days and then allowed to recover for 5 days. D denotes the time course day. Different letters indicate significant differences as determined by a post-hoc Tukey’s HSD test at *p* < 0.05.

**Figure 7:**
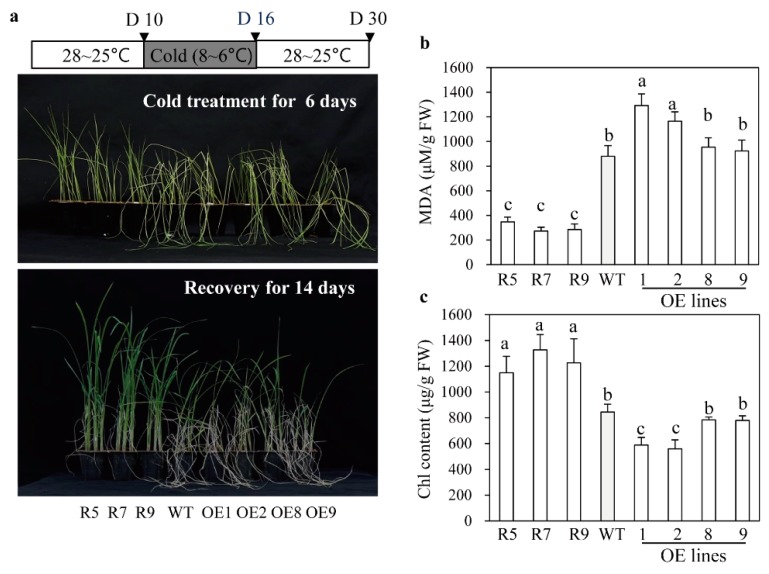
Response to cold treatment. (**a**) Rice seedlings after cold treatment and recovery from cold stress. (**b**) MDA content. (**c**) Chlorophyll content. Ten-day-old rice seedlings were exposed to cold stress at 6–8 °C for 6 days and then allowed to recover for 14 days at 25–28 °C. D denotes the time course day. Different letters indicate significant differences as determined by a post-hoc Tukey’s HSD test at *p* < 0.05.

**Figure 8:**
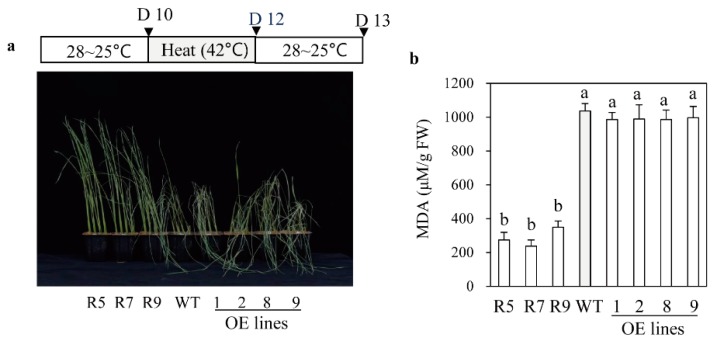
Response to heat treatment. (**a**) Seedlings after recovery from heat stress. (**b**) MDA content. Ten-day-old rice seedlings were challenged with heat stress at 42°C for 2 days and then allowed to recover for 1 day. Different letters indicate significant differences as determined by a post-hoc Tukey’s HSD test at *p* < 0.05.

In addition to cadmium tolerance, the *snat2* plants also exhibited increased salt stress tolerance, whereas the *SNAT2* OE lines were more susceptible to salt stress, as indicated by their MDA levels (Figure 6a,b). Accordingly, the average survival rate of *snat2* plants under salt stress was 55%, while those of the wild type plants and the *SNAT2* OE lines were only 30% and less than 20%, respectively (Figure 6c). In response to cold stress, the survival rate of *snat2* rice was higher than that of the wild type based on MDA and chlorophyll contents (Figure 7), whereas the *SNAT2* OE lines exhibited lower survival rates than the wild type. Heat stress produced high MDA levels in the wild type, whereas the MDA levels in *snat2* rice were three times lower on average than in the wild type (Figure 8). In contrast to salt and cold stress, the *SNAT2* OE lines showed similar heat stress response relative to the wild type. Collectively, these results showed that melatonin-deficient *snat2* rice exhibited enhanced tolerance to multiple abiotic stress conditions, consistent with the results of previous studies of plants with reduced BR and GA levels [41,42].

### 2.6. Enhanced Tolerance to Salt and Heat Stress in Other Types of Melatonin-Deficient Rice

**Figure 9:**
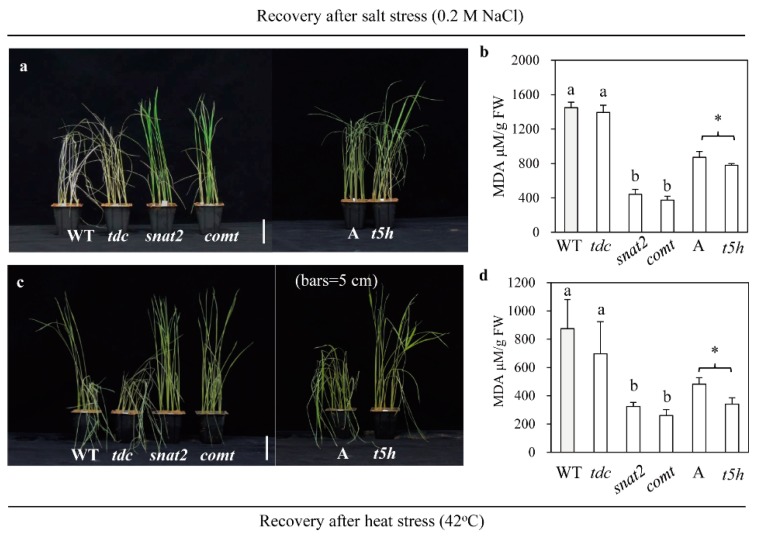
Stress tolerance of melatonin-deficient rice mutant lines. (**a**) Salt-treated rice seedlings. (**b**) MDA levels after salt treatment. (**c**) Heat-treated rice seedlings. (**d**) MDA levels after heat treatment. Methods for salt and heat treatment are described in Figure 6 and Figure 8, respectively. *tdc*, *TD*C suppression rice; *snat2*, *SNAT2* suppression R7 rice; *comt*, *COMT* suppression rice; A, Ashahi rice (wild type of *t5h* mutant rice); *t5*h, *T5H* mutant rice. Different letters and asterisks indicate significant differences as determined by a post-hoc Tukey’s HSD test at *p* < 0.05.

To determine whether other genetic cultivars with melatonin deficiency also confer tolerance to abiotic stress in rice seedlings, we employed two RNAi rice lines and one mutant rice line known to produce lower levels of melatonin than the wild type. The *tdc* RNAi rice lines did not exhibit altered BR levels, whereas *comt* RNAi rice exhibited reduced BR levels [23]. Lower levels of melatonin and BR are produced in *t5h* rice than in the wild type [23]. Seedlings of the *snat2*, *comt*, and *t5h* melatonin-deficient lines exhibited significant tolerance to both salt and heat stress, but no tolerance was observed in the *tdc* rice, as indicated by their MDA levels (Figure 9). This difference on abiotic stress responses was due to the lack of reduced BR levels in *tdc* rice, in contrast with the reduced BR levels in the other types of melatonin-deficient rice relative to the wild type [23]. Based on the above observations, we conclude that endogenous melatonin deficiency is closely associated with enhanced abiotic stress tolerance only when melatonin deficiency is coupled with reduced BR levels.

## 3. Discussion

Melatonin has long been considered to be a positive regulator in plants, enhancing tolerance to various abiotic and biotic stresses including cold, heat, salt, and drought [4]. Various pharmacological studies have shown that exogenous melatonin treatments with concentrations ranging from 0.1 μM to 1 mM reduced damage caused by a number of abiotic stresses, as evidenced by decreased levels of reactive oxygen species (ROS), MDA, and electrolyte leakage, as well as increases in antioxidants and antioxidative enzymes [44,45,46]. The induction of tolerance to adverse environments by exogenous melatonin application appears to be due to the combined effects of the potent antioxidative activity of melatonin itself [6] and its role as a pivotal signaling molecule [47,48,49]. In addition, many molecular and genetic experiments have confirmed the results of the pharmacological studies mentioned above. For instance, gain of function analyses showed that plants overexpressing melatonin biosynthetic genes exhibited increased tolerance to cold [50], UV-B light [51], herbicide [52], drought [53,54,55], heat [56], salt [57], cadmium [32,58,59,60], and continuous light [61]. Complementary to the gain of function analyses, loss of function analyses via either suppression or knockout of melatonin biosynthesis demonstrated increased sensitivity to tunicamycin [49], high light [62], salt [63], senescence [22], and pathogens [64]. All of these data strongly suggest that melatonin plays a positive role in stress tolerance in plants.

In marked contrast to previous results, we found a paradoxical effect of melatonin. Even though the *snat2* rice plants produced low melatonin levels compared to the wild type, they proved to be tolerant to various abiotic stresses including cadmium, cold, salt, and heat, possibly because the *snat2* rice produced lower levels of BR than the wild type [21].

BR is a well-known plant hormone that controls multiple physiological processes throughout the entire life cycle of the plant, including seed germination, cell elongation, and skotomorphogenesis, as well as defense responses against various abiotic and biotic stresses [27]. Like melatonin, BR is synthesized in all plant cells without transport over long distances. Thus, its de novo synthesis and turnover are key factors that regulate BR levels. It is known that exogenous application of BR to plants improves protection against many adverse conditions such as cold, salt, drought, heat, and heavy metal stresses, by increasing the activities of antioxidant enzymes such as catalase, and the levels of glutathione and proline [28,65]. Interestingly, BR treatment also confers resistance to a broad range of diseases in tobacco and rice, in conjunction with increased yield, via its growth-promoting activity [28,66]. In contrast to exogenous BR treatment, increasing the level of endogenous BR through the ectopic OE of BR biosynthetic genes results in decreased tolerance to stress, while BR-deficient mutants exhibit increased drought tolerance via increased sensitivity to abscisic acid (ABA) [31]. A BR receptor mutant (*bri1*) exhibited cold tolerance, while *BRI1*-overexpressing *Arabidopsis* plants were more sensitive to cold stress, indicative of an inverse relationship between BR and stress tolerance [29,30].

Recently, a more detailed analysis of BR function showed that BR regulates cell elongation by modulating GA metabolism in rice [42]. Under physiological conditions, BR promotes GA production, while GA levels are reduced in BR-deficient mutants. However, treatment with excessive BR reduces GA levels through upregulation of GA inactivation genes, resulting in growth inhibition. By contrast, application of GA-inhibitors retards plant growth and increases tolerance to many abiotic stresses, including drought. Accordingly, GA application reverses enhanced stress tolerance in GA-inhibitor treated plants and GA-deficient mutants [4], suggesting that GA application will cause the plants to be less tolerant to various abiotic stresses. In addition, the suppression of *IAA6* in the *snat2* was ascribed to BR decrease because *IAA6* expression was dependent on BR [67]. In contrast, the similar suppression patterns of *IAA6* in the OE lines without BR decrease remained unclear.

**Figure 10:**
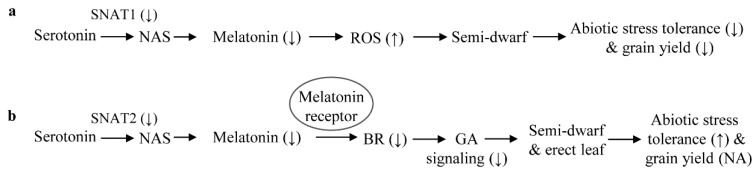
A proposed mechanism for melatonin-mediated abiotic stress tolerance. Two different mechanisms underlie melatonin-mediated plant responses. One mechanism is a BR-independent pathway (**a**). This pathway is associated directly with the antioxidant activity of melatonin. Melatonin-deficient rice plants, such as *snat1*, produce increased levels of reactive oxygen species (ROS), which in turn inhibit plant growth and dampen abiotic stress tolerance. The other mechanism is a BR-dependent pathway (**b**). This pathway involves melatonin as a signaling molecule, possibly via a melatonin receptor [67]. Melatonin-deficient rice plants, such as *snat2*, exhibit decreased BR levels in conjunction with increased GA insensitivity, leading to enhanced abiotic stress tolerance. The basis of differential receptor recognition of melatonin deficiency caused by either *SNAT1* or *SNAT*2 remains unclear. NAS, *N*-acetylserotonin; NA, not altered.

Based on the above observations, it is clear that melatonin deficiency leads to decreased BR levels, which in turn reduce GA sensitivity resulting in increased tolerance to multiple abiotic stresses such as cadmium, cold, heat, and salt (Figure 5, Figure 6, Figure 7 and Figure 8). In this study, our examination on the effects of decreased BR levels were not limited to the melatonin-deficient *snat2* rice; we also examined the melatonin-deficient *t5h* and *comt* rice lines, which also produced lower BR levels than the wild type [23]. All three of these rice lines were BR-deficient due to decreased melatonin levels, and exhibited enhanced tolerance to multiple abiotic stresses. However, the *snat1* and *tdc* mutants did not exhibit suppressed BR levels even though both produced less melatonin than the wild type. Thus, it is tempting to speculate that melatonin exerts physiological functions in a BR-dependent or -independent manner in plants, although the detailed mechanism remains to be determined (Figure 10). For example, *SNAT1* is involved in melatonin synthesis independent of BR synthesis, and its suppression leads to decreased melatonin levels resulting in decreased tolerance to abiotic stress and reduced yield, possibly due to increased ROS levels (Figure 10a). *TDC* functions in a similar manner to *SNAT1* in terms of melatonin synthesis and abiotic stress responses. Conversely, *snat2* resulted in decreased melatonin in conjunction with decreased BR, which conferred tolerance to cadmium, cold, salt, and heat stress (Figure 10b). Like *SNAT2*, the suppression of other melatonin biosynthetic genes, including *T5H* and *COMT*, also conferred tolerance to cold and heat stress in a similar way to BR decrease [23]. This suppression of BR levels in response to decreased melatonin appears to be mediated by a melatonin receptor [68], but its direct role remains elusive. In particular, the grain phenotypes of *snat2* rice were identical to those of the BR-deficient and BR-signaling mutants reported thus far, confirming that decreased melatonin is coupled to decreased BR even at the reproductive stage [34,36].

## 4. Materials and Methods

### 4.1. Plant Materials

Transgenic rice plants in which expression of the rice *SNAT2* gene was downregulated by RNA interference (RNAi) or overexpressed were generated in a previous study [21]. *TDC* RNAi, *T5H* knockout mutant, and *COMT* RNAi rice plants were described previously [23]. *TDC* RNAi and *COMT* RNAi rice plants were generated by *Agrobacterium*-mediated transformation using the same pTCK303 binary vector described previously [23,69]. *T5H* mutant rice plants lacking a functional *T5H* gene were screened from ^137^Cs-irradiated Ashahi seeds and obtained from the National Institute of Agrobiological Sciences (NIAS) GeneBank (http://www.gene.affrc.go.jp/plant/).

### 4.2. Plant Growth Conditions

Wild-type and transgenic rice (*Oryza sativa*) seeds were soaked in distilled water (DW), and the germinated seeds were transferred onto soil. Plants were grown in a culture room at 28 °C/24 °C (day/night) with a 14-h light/10-h dark cycle, or in a paddy field at the Chonnam National University (53 m a.s.l.; 35°09′ N and 126°54′ W), Gwangju, Korea. The angles of the lamina joint of the second leaf were measured in 12-day-old rice seedlings.

### 4.3. Semi-Quantitative Reverse Transcription–Polymerase Chain Reaction (RT-PCR) Analysis

The NucleoSpin RNA Plant Kit (Macherey-Nagel, Düren, Germany) was used to extract total RNA. First-strand cDNA was synthesized from 2 μg of total RNA using MG MMLV Reverse Transcriptase (MGmed, Inc., Seoul, Korea) and an oligo dT18 primer at 42 °C for 1 h. The rice ubiquitin-5 (*UBQ5*) gene was used as a loading control. PCR was carried out as described previously [19]. The primers used for the various genes were as follows: *GA3ox2* (forward 5′-TCCTCCTTCTTCTCCAAGCTCAT-3′; reverse 5′-GAAACTCCTCCATCACGTCACA-3′), *GA2ox3* (forward 5′-TGGTGGCCAACAGCCTAAAG-3′; reverse 5′-TGGTGCAATCCTCTGTGCTAAC-3′), *DWARF4* (forward 5′-GTGCTGCCATTCTCGGAGTAATAG-3′; reverse 5′-CTCAGCAAGAGGTCCAGGATTTGC-3′), *IAA6* (forward 5′-CACCATGGAAGAAGGGTCCAACAAAA-3′; reverse 5′-TTAGACCCTAGCAGTAGCTCCA-3′), and *UBQ5* (forward 5′-CCGACTACAACATCCAGAAGGAG-3′; reverse 5′-AACAGGAGCCTACGCCTAAGC-3′). GenBank accession numbers are AB056519 (*GA3ox2*), AK101713 (*GA2ox3*), Os03g12660 (*DWARF4*), Os01g0741900 (*IAA6*), and AK061988 (*UBQ5*).

### 4.4. Hormone Treatment

Gibberellic acid (GA_3_) and paclobutrazol (PAC) (Sigma-Aldrich, St. Louis, MO, USA) were dissolved in ethanol. An identical volume of the blank solvent (ethanol) was used as a mock treatment. To detect GA sensitivity, sterilized seeds were first soaked in 150 μM PAC for 2 days to inhibit endogenous GA biosynthesis, and then germinated seeds were grown in DW with various concentrations of GA3. Seedling lengths were measured after 7 days of growth.

### 4.5. Melatonin Quantification

Rice samples (100 mg) were frozen in liquid nitrogen and pulverized to a powder using TissueLyser II (Qiagen, Tokyo, Japan), and extracted with 1 mL of chloroform at room temperature as described previously [70]. In brief, the chloroform extracts (200 µL) were completely evaporated and dissolved in 0.1 mL of 40% methanol, and 10-µL aliquots were subjected to high performance liquid chromatography coupled with a fluorescence detector system (Waters, Milford, MA, USA). Melatonin was detected at 280 nm (excitation) and 348 nm (emission). The detection limit and recovery rate of melatonin were approximately 0.25 ng/g FW and 90%, respectively, using this analytical method. All measurements were made in triplicate.

### 4.6. Germination Assay

Wild-type and transgenic rice seeds were sterilized with 100% ethanol for 1 min followed by 2% sodium hypochlorite for 5 min. The seeds were then rinsed three times in DW for 30 min. Thirty-five seeds were placed in 3 mL of DW in six-well plates. Germination tests were carried out in a culture room at 28 °C/24 °C (day/night) with a 14-h light/10-h dark cycle. A seed was considered to be germinated when the seed coat ruptured and the radicle was emerged and >1 mm in length. Each treatment was repeated three times.

### 4.7. Salt, Cold, Heat, and Cadmium Stress Tolerance Assays

Wild-type and transgenic plants were grown in a culture room with 30 μmol photons·m^−2^·s^−1^ at 26 °C/24 °C (day/night) under a 14-h light/10-h dark photoperiod. For salt stress assays, 10-day-old seedlings grown in pots were irrigated with 200 mM NaCl for 6 days and then allowed to recover for another 5 days. For cold stress assays, 10-day-old seedlings grown in pots were exposed to 6–8 °C for 6 days and then allowed to recover for another 14 days at 25–28 °C. For heat stress assays, 10-day-old seedlings grown in pots were exposed to 42 °C for 2 days and then allowed to recover for 1 day at 25–28 °C. For cadmium stress assays, 7-day-old seedlings were placed in 50-mL polypropylene conical tubes containing 30 mL of 0.5 mM CdCl_2_ and incubated for 3 days under long day (LD) conditions (14-h light/10-h-dark photoperiod with 60 μmol photons·m^−2^·s^−1^).

### 4.8. Determination of Chlorophyll and Malondialdehyde (MDA) Contents

Rice samples were frozen in liquid nitrogen and ground to a powder using TissueLyser II (Qiagen). Chlorophyll was extracted from the powder (100 mg) in 1 mL of 0.1 M NH_4_OH (containing 80% acetone). Chlorophyll concentrations were determined at wavelengths of 647, 644, and 750 nm using a spectrophotometer (Optizen Pop-Bio; Mecasys, Daejeon, Korea). For MDA quantification, the powder (50 mg) was extracted with 1.5 mL of reaction buffer containing 0.5% thiobarbituric acid (TBA) and 20% trichloroacetic acid (TCA). The samples were centrifuged for 15 min at 12,000× *g*, and the resulting supernatants were collected for measurement. The supernatant was boiled at 95 °C for 25 min and placed in ice for 5 min. MDA content was determined at 440, 532, and 600 nm using a spectrophotometer (Optizen Pop-Bio) and a molar extinction coefficient of 156 nM·cm^−1^.

### 4.9. Quantification of Bioactive BRs and GA

Levels of bioactive BRs and GA were quantified with a quantitative sandwich enzyme-linked immunosorbent assay (ELISA) kit (MyBioSource, San Diego, CA, USA). Briefly, 10-day-old rice seedlings (100 mg) were pulverized in liquid nitrogen and homogenized in 1 mL of phosphate-buffered saline (PBS) (pH 7.5; Sigma-Aldrich). The resulting samples were centrifuged for 20 min at 5000× *g*, and the supernatants were collected for measurement. The supernatants (100 μL) were subjected to ELISA according to the manufacturer’s instructions (MyBioSource). Optical density was measured at 450 nm using a microplate reader (Molecular Devices, Sunnyvale, CA, USA), as described previously [71].

### 4.10. Statistical Analysis

Data were analyzed by analysis of variance using IBM SPSS Statistics software (ver. 23.0; IBM Corp., Armonk, NY, USA). Means with different letters or asterisks indicate significantly different values at *p* < 0.05 according to a post-hoc Tukey’s honestly significant difference (HSD) test or least significance difference (LSD) test. All data are presented as means ± standard deviation.
